# Hepatitis B reactivation in cancer patients receiving immune checkpoint inhibitors: a systematic review and meta-analysis

**DOI:** 10.1186/s40249-023-01128-6

**Published:** 2023-09-22

**Authors:** Zhengzheng Xia, Jianyu Zhang, Wenjun Chen, Haiyan Zhou, Di Du, Kongcai Zhu, Hui Chen, Jun Meng, Jun Yang

**Affiliations:** 1 Department of Pharmacy, National Cancer Center/National Clinical Research Center for Cancer/Cancer Hospital, Chinese Academy of Medical Sciences and Peking Union Medical College, Beijing, China; 2 Department of Pharmacy, National Cancer Center/National Clinical Research Center for Cancer/Cancer Hospital and Shenzhen Hospital, Chinese Academy of Medical Sciences and Peking Union Medical College, Shenzhen, China; 3 Department of Pharmacy, Hubei Cancer Hospital, Tongji Medical College, Huazhong University of Science and Technology, Wuhan, China; 4 Department of Pharmacy, Beijing Youan Hospital, Capital Medical University, Beijing, China; 5 Department of Pharmacy, Tangshan Central Hospital, Tangshan, China

**Keywords:** Hepatitis B virus reactivation, Immune checkpoint inhibitors, Cancer, Systematic review, Meta-analysis, Safety

## Abstract

**Background:**

Immunotherapy shows promise as a treatment option for various cancers. However, there is growing concern over potential complications from hepatitis B virus (HBV) reactivation after checkpoint blockade immunotherapy. Although most of the previous clinical trials on immune checkpoint inhibitors (ICIs) excluded patients with HBV, a few case reports and retrospective studies of HBV reactivation have been published. The aim of this study is to assess the risk of hepatitis B virus reactivation (HBVr) in patients receiving ICIs for advanced cancer.

**Methods:**

English and Chinese language literature published prior to April 30, 2023, was searched in PubMed, EMBASE, Web of Science, Cochrane, SinoMed, CNKI and Wanfang Data for studies reporting HBVr rates in cancer patients treated with ICIs. A pooled risk estimate was calculated for HBVr rates with 95% confidence intervals (*CI*).

**Results:**

Data from 34 studies including 7126 patients were retrieved and analyzed. The pooled HBVr rate in cancer patients treated with ICIs was 1.3% (*I*^2^ = 90.44%, 95% *CI*: 0.2–2.9%, *P* < 0.001). Subgroup analysis revealed that patients diagnosed with hepatocellular carcinoma (HCC), HBV carriers, and patients from Asian regions or in developing countries have a higher rate of HBVr.

**Conclusions:**

Our meta-analysis demonstrated a low risk of HBVr in patients treated with ICIs for advanced cancer. ICI treatment may be safely used in patients with existing HBV infection or chronic hepatitis B, accompanied by regular monitoring and appropriate antiviral prophylaxis if necessary.

**Graphical Abstract:**

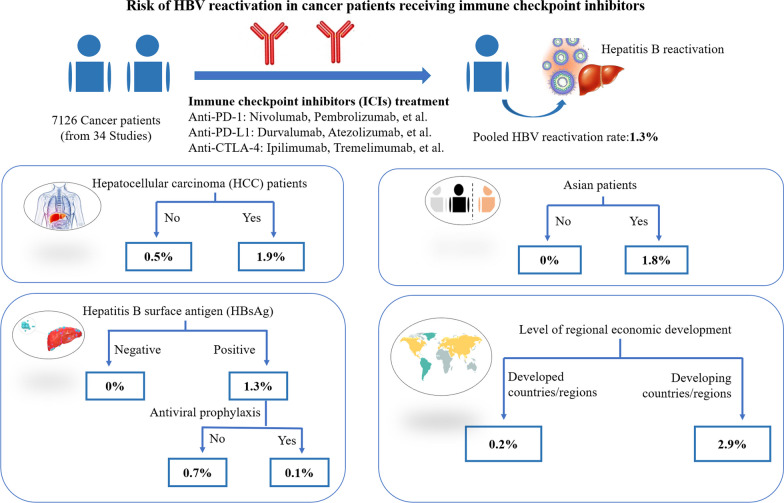

**Supplementary Information:**

The online version contains supplementary material available at 10.1186/s40249-023-01128-6.

## Background

Immunotherapy has emerged as a popular therapeutic approach for cancer patients in recent years. However, the issue of hepatitis B virus reactivation (HBVr) has become a matter of increasing concern among some patients. Chronic hepatitis B represents a significant public health problem worldwide, with a high prevalence in East Asia. There are approximately 316 million hepatitis B surface antigen (HBsAg)-seropositive patients, and an estimated 1.5 million new infections annually, particularly in developing and impoverished countries [1]. Given the large number of HBV carriers, many cancer patients also have concurrent hepatitis virus infection, which presents a considerable challenge.

Immune checkpoint inhibitors (ICIs), which target programmed cell death protein 1 (PD-1), programmed cell death ligand 1 (PD-L1) and cytotoxic T lymphocyte antigen 4 (CTLA-4), have revolutionized cancer therapy. As an increasing number of patients are exposed to these agents, the population eligible for ICI treatment continues to expand. However, patients with special clinicopathological characteristics, such as those with viral hepatitis, have often been excluded from clinical trials in the past, leading to a lack of efficacy and safety data [1–3].

Recent studies have shown that HBVr may occur in chronic hepatitis B (HBsAg-positive) patients or even in patients with resolved HBV (HBsAg-negative/HBcAb-positive) infection during immunotherapy [4–6], which might cause a potentially fatal complication for cancer patients. Furthermore, HBVr could also cause interruption of antineoplastic therapy and impact overall survival. As the rate of HBVr and potential risk factors for HBVr in patients treated with ICI-based therapy remain undefined, there is a lack of consensus among various organizations regarding the optimal management strategies for this patient population [7, 8].

Evaluating the potential risk of viral reactivation during ICI-based therapy could assist medical professionals in assessing the suitability of immunotherapy and may be useful for budget and cost-effectiveness analysis in pharmacoeconomics studies. Herein, we performed a meta-analysis to estimate the rate of HBVr in chronic carriers of HBsAg and patients with resolved hepatitis B who received ICI-based therapy for advanced cancer.

## Methods

This systematic review and meta-analysis was performed in accordance with the Preferred Reporting Items for Systematic Reviews and Meta-Analyses (PRISMA) statement [9]. This study was registered in PROSPERO with registration number CRD42022330949.

### Retrieval of studies

To retrieve relevant studies, we conducted a comprehensive search of multiple databases, including PubMed, EMBASE, Web of Science and the Cochrane databases, covering literature prior to April 30, 2023, limited to the English language. Additionally, we searched the SinoMed (http://www.sinomed.ac.cn/index.jsp), CNKI (https://www.cnki.net/) and Wanfang database (https://wanfangdata.com.cn/) prior to April 30, 2023, limited to the Chinese language. Our search terms included cancer, tumor, ICIs (anti-PD-1, anti-PD-L1, anti-CTLA-4), specific ICI names (nivolumab, pembrolizumab, atezolizumab, durvalumab, avelumab, ipilimumab, sintilimab, etc.), and relevant terms related to HBV flare or reactivation. The detailed search strategy is provided in Additional file 1: Table S1.

The studies that were included in this meta-analysis had to meet the following criteria: (1) the study was conducted on human subjects and was either interventional or observational; (2) the patients were diagnosed with a solid tumor and had received at least one cycle of ICI therapy; and (3) the study reported complete outcomes that measured the incidence of HBVr.

Studies published as case reports or series, editorials, comments, letters and review articles were excluded. Given the potential influence of other types of hepatitis, patients co-infected with hepatitis C virus (HCV), hepatitis A virus (HAV), hepatitis D virus (HDV), or hepatitis E virus (HEV) were excluded. Additionally, as the presence of active HBV replication may further exacerbate HIV-induced immune deficiency, patients co-infected with HBV and HIV were also excluded from the literature review. Overlapping patient data were comprehensively reviewed, prioritizing the study with the most useful and detailed information [10–16].

The initial screening of citations was based on the evaluation of their titles and abstracts. Subsequently, the full texts of relevant citations were further assessed to determine their eligibility for inclusion in the systematic review. Any conflicts among the researchers regarding study selection were resolved through discussion, and by referring back to the original article until a consensus was reached between all authors.

### Quality evaluation

We used the Newcastle‒Ottawa Scale (NOS) to assess the quality of each study based on patient selection, comparability of groups, and assessment of outcome [17]. Studies with less than six stars were considered relatively low quality and were excluded. Two independent investigators evaluated the risk of bias, and disagreements were resolved through discussion until a consensus was reached.

### Data extraction

Two independent investigators screened the titles and abstracts for eligible studies according to the inclusion criteria, and any discrepancies were resolved through discussion among all authors. We extracted the following information from the eligible articles: country/region, author, publication year, study type, number of patients, median age, HBV infection, tumor type, ICIs type, HBVr status, and use of antiviral drugs. For missing data, we contacted the authors of the studies for unreported data or additional details.

### Outcome measures

The primary outcome for this review was the rate of HBVr in patients with preexisting HBV infection or chronic hepatitis B who received ICI treatment for malignancies. HBVr was defined based on the American Association for the Study of Liver Diseases (AASLD) 2018 hepatitis B guideline [18], the American Society of Clinical Oncology (ASCO) 2020 HBV guidance [8] and other references [19], which was a relatively loose definition to include those patients with mild HBV increase and/or HBsAg reappearance. It could be interpreted as follows: for HBsAg-positive patients, (1) a tenfold increase in HBV DNA from baseline levels; (2) a 2-log (100-fold) increase in HBV DNA compared with baseline levels; (3) HBV DNA ≥ 3 log (1000) IU/ml in a patient with previously undetectable levels (given that HBV-DNA levels fluctuate); or (4) HBV DNA ≥ 4 log (10,000) IU/ml if the baseline level was not available; for HBsAg-negative patients, (1) detectable HBV DNA; or (2) HBsAg seroreversion (reappearance of HBsAg).

### Statistical analysis

The probabilities of HBVr were estimated using a random-effects model [20]. Cochran chi-square heterogeneity was adopted to determine whether there was statistically significant heterogeneity in the pooled estimates. The* I*^2^ statistic was calculated as a measure of the degree of heterogeneity among selected studies, where *I*^*2*^ values of 25%, 50% and 75% were considered low, moderate and high degrees of heterogeneity, respectively. Significant heterogeneity was investigated by subgroup analysis. Publication bias assessment was not performed because the outcome measure was the single-group rate. All statistical analyses were conducted using Stata software (Version 16.0, Stata Corporation, College Station, TX, USA).

## Results

### Characteristics of the included studies

**Fig. 1:**
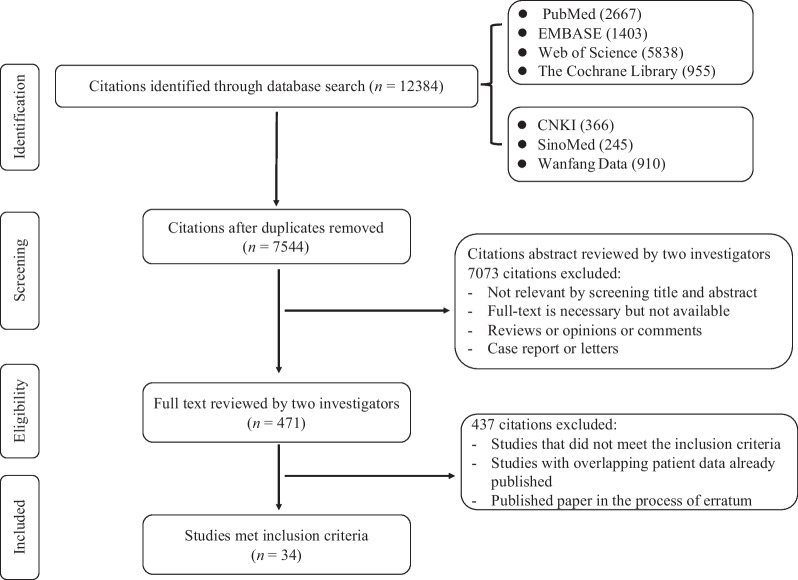
Flowchart of study selection procedure

As illustrated in the flowchart of the literature search strategy (Fig. 1), a total of 12,384 articles were retrieved from the PubMed, EMBASE, Web of Science, Cochrane, SinoMed, CNKI and Wanfang databases. After removing duplicates and scrutinizing abstracts, 471 potentially eligible studies were identified, of which 34 studies with a total number of 7126 patients were ultimately included in this meta-analysis (Table 1). HBVr events were reported in 16 of the included studies; but not in the remaining 18 studies. These selected studies were published between 2018 and 2023. In terms of geographical origin, 25 studies were conducted in Asia (17 from China, 3 from Singapore, 3 from the Republic of Korea, and 2 from Japan), 5 studies were from North America (United States), 3 were from Oceania (2 from Australia and 1 from New Zealand) and 1 was from Europe (Italy).

**Table 1:** Basic characteristics of included studies

First author (publication year)	Study design	Study country	Total patients	Name of ICIs	Tumor type	Anti-neoplastic agent combined with ICIs	HBV Reactivation rate (total)	HCC (Yes vs No)	Reactivation rate (HCC vs non-HCC)	HBsAg (+ vs −)	HBV Reactivation rate (HBsAg + vs HBsAg-)	Median follow-up time
Zhu et al. (2018) [30]	Clinical trial	United States	103	Pembrolizumab	HCC	ICI monotherapy	0/103 (0%)	103/0	0 vs 0	22/81	0 vs 0	12 months
Tio et al. (2018) [31]	Retrospective	Australia	14	Atezolizumab, ipilimumab, nivolumab, pembrolizumab	MM, HCC, GC, UTUC, GBM	ICI monotherapy	0/14 (0%)	1/13	0 vs 0	14/0	0 vs 0	N.A
Yau et al. (2019) [32]	Clinical trial	China	105	Nivolumab	HCC	ICI monotherapy	10/105 (9.52%)	105/0	9.52% vs 0	N.A	N.A	31.6 months
Finn et al. (2019) [33]	Clinical trial	United States	72	Pembrolizumab	HCC	ICI monotherapy	0/72 (0%)	72/0	0 vs 0	N.A	N.A	13.8 months
Gane et al. (2019) [28]	Clinical trial	New Zealand	14	Nivolumab	N.A	ICI monotherapy	0/14 (0%)	N.A	N.A	14/0	0 vs 0	24 weeks
Shah et al. (2019) [34]	Retrospective	United States	15	Atezolizumab, avelumab, durvalumab, ipilimumab nivolumab, pembrolizumab	HCC, NSCLC	ICI ± chemotherapy	0/15 (0%)	N.A	N.A	8/7	0 vs 0	N.A
Zhang et al. (2019) [35]	Retrospective	China	101	Atezolizumab, camrelizumab, ipilimumab, nivolumab, pembrolizumab, sintilimab, toripalimab	NPC, HCC, MM	ICI ± chemotherapy or apatinib or bevacizumab or cetuximab or nimotuzumab or osimertinib or regorafenib or sunitinib	6/101 (5.94%)	28/73	3.57% vs 6.85%	101/0	5.94% vs 0	N.A
Qin et al. (2020) [36]	Clinical trial	China	180	Camrelizumab	HCC	ICI monotherapy	0/180 (0%)	180//0	0 vs 0	180/0	0 vs 0	12.5 months
Pertejo-Fernandez et al. (2020) [37]	Retrospective	United States	14	Atezolizumab, durvalumab, ipilimumab, nivolumab, pembrolizumab	NSCLC	ICI ± chemotherapy or ICIs combination therapy	0/14 (0%)	0/14	0 vs 0	2/12	0 vs 0	N.A
Lee et al. (2020) [38]	Retrospective	China	60	Nivolumab, pembrolizumab	HCC	ICI + TKIs not specified	1/60 (1.67%)	60/0	1.67% vs 0	N.A	N.A	6.6 months
Byeon et al. (2020) [39]	Retrospective	The Republic of Korea	32	Nivolumab, pembrolizumab	NSCLC	ICI monotherapy	3/32 (9.38%)	0/32	0 vs 9.38%	16/16	18.75% vs 0	6 months
Chan et al. (2020) [40]	Retrospective	Singapore	42	Atezolizumab, durvalumab nivolumab, pembrolizumab	NSCLC	ICI ± chemotherapy	1/42 (2.38%)	0/42	0 vs 2.38%	8/34	12.5% vs 0	6 months
Ng et al. (2020) [41]	Retrospective	Singapore	62	Not specified	HCC	IC I ± chemotherapy or targeted agent not specified	6/62 (9.68%)	62/0	9.68% vs 0	55/7	9.09% vs 14.29%	13.8 months
Chen et al. (2020) [42]	Retrospective	China	70	Camrelizumab, sintilimab, toripalimab	HCC	ICI + lenvatinib or sorafenib or apatinib	0/70 (0%)	70/0	0 vs 0	70/0	0 vs 0	44.7 weeks
Saw et al. (2020) [43]	Retrospective	Australia	127	Not specified	N.A	N.A	0/127 (0%)	N.A	N.A	0/127	0 vs 0	N.A
Zhong et al. (2021) [44]	Retrospective	China	15	Camrelizumab, nivolumab, pembrolizumab, sintilimab, toripalimab	HCC	IC I ± chemotherapy or anti-neoplastic agent not specified	0/15 (0%)	4/11	0 vs 0	15/0	0 vs 0	6 months
Xu et al. (2021) [45]	Retrospective	China	17	Camrelizumab, nivolumab, pembrolizumab, sintilimab, tislelizumab, triprizumab	LC	ICI ± chemotherapy	0/17 (0%)	0/17	0 vs 0	N.A	N.A	7.5 months
Wang et al. (2021) [46]	Retrospective	China	182	Atezolizumab camrelizumab, durvalumab, nivolumab, pembrolizumab, sintilimab, tislelizumab, toripalimab	HCC	ICI + apatinib or bevacizumab or lenvatini or regorafenib or sorafenib	8/182 (4.40%)	182/0	4.40% vs 0	182/0	4.40% vs 0	8 months
Wong et al. (2021) [6]	Retrospective	China	990	Atezolizumab, avelumab, durvalumab ipilimumab, nivolumab, pembrolizumab, spartalizumab, tremelimumab	HCC	ICI monotherapy or ICIs combination therapy	3/990 (0.30%)	N.A	N.A	397/ 593	0.50% vs 0.17%	6.9 months
He et al. (2021) [47]	Retrospective	China	202	Camrelizumab nivolumab, pembrolizumab, sintilimab, toripalimab,	HCC	ICI + lenvatinib or regorafenib or sorafenib	7/202 (3.47%)	202/0	3.47% vs 0	202/0	3.47% vs 0	6 months
Yoo et al. (2021) [48]	Retrospective	The Republic of Korea	3465	Atezolizumab, avelumab, durvalumab, ipilimumab, nivolumab, pembrolizumab, tremelimumab	LC, HPBC, GC, UC	ICI monotherapy or ICIs combination therapy	5/3465 (0.14%)	524/2941	0.38% vs 0.10%	511/ 2954	0.98% vs 0	6 months
Lee et al. (2021) [49]	Clinical trial	Singapore	36	Nivolumab	HCC	ICI monotherapy	0/36 (0%)	36/0	0 vs 0	22/14	0 vs 0	24.8 months
Zhang et al. (2021) [50]	Retrospective	China	62	Atezolizumab, camrelizumab nivolumab, pembrolizumab	NSCLC	ICI monotherapy	1/62 (1.61%)	62/0	1.61% vs 0	10/52	1% vs 0	28.4 months
Lee et al. (2021) [51]	Clinical trial	The Republic of Korea	26	Avelumab	HCC	ICI monotherapy	0/26 (0%)	26/0	0 vs 0	26/0	0 vs 0	13.9 months
Zhao et al. (2022) [52]	Retrospective	China	60	Not specified	NSCLC	ICI ± chemotherapy or/and bevacizumab	0/60 (0%)	0/60	0 vs 0	3/57	0 vs 0	6.49 months
Hagiwara et al. (2022) [53]	Retrospective	Japan	166	Not specified	HCC	ICI ± chemotherapy or targeted agent not specified	1/166 (0.6%)	28/138	3.57% vs 0	24/142	4.17% vs 0	48 weeks
Cheng et al. (2022) [54]	Retrospective	China	77	Camrelizumab nivolumab, pembrolizumab, teriprizumab, toripalimab	CRC	ICI ± chemotherapy or TKIs not specified	0/77 (0%)	0/77	0 vs 0	20/57	0 vs 0	N.A
Nakabori et al. (2022) [55]	Retrospective	Japan	266	Atezolizumab, avelumab, durvalumab, ipilimumab, nivolumab, pembrolizumab	N.A	ICI ± chemotherapy or axitinib or bevacizumab	0/266 (0%)	N.A	N.A	8/258	0 vs 0	N.A
Shun et al. (2022) [56]	Clinical trial	China	17	Nivolumab	NSCLC	ICI monotherapy	3/17 (17.65%)	0/17	0 vs 17.65%	17/0	17.65% vs 0	37.6 months
Hu et al. (2022) [57]	Retrospective	China	70	Atezolizumab, tislelizumab, other anti-PD-1/L1 not specified	HCC	ICI + apatinib or bevacizumab or lenvatinib or regorafenib or sorafenib	2/70 (2.86%)	70/0	2.86% vs 0	70/0	2.86% vs 0	N.A
Lei et al. (2023) [58]	Retrospective	China	203	Atezolizumab, camrelizumab, nivolumab, pembrolizumab, sintilimab, tislelizumab, toripalimab	HCC	ICI + chemotherapy or lenvatinib or sorafenib	61/203 (30.05%)	203/0	30.05% vs 0	203 vs 0	30.05% vs 0	5 months
Lasagna et al. (2023) [59]	Retrospective	Italy	150	Atezolizumab, nivolumab, pembrolizumab	MM, RCC, HNC, other tumor not specified	ICI ± chemotherapy	0/150 (0%)	0/150	0 vs 0	0 vs 150	0 vs 0	12 months
Nardo et al. (2023) [60]	Retrospective	United States	10	Anti-PD-1 ± Anti-CTLA-4	N.A	ICI ± chemotherapy or bevacizumab	0/10 (0%)	N.A	N.A	10/0	0 vs 0	33 months
Chen et al. (2023) [61]	Retrospective	China	101	ICIs not specified	HCC	ICI + TKIs not specified	5/101 (5.0%)	101/0	5.0% vs 0	N.A	N.A	11.68 months

*TKIs* tyrosine kinase inhibitors, *MM* malignant melanoma, *GBM* glioblastoma multiforme, *HCC* hepatocellular carcinoma, *UTUC* upper tract urothelial carcinoma, *NSCLC* non-small cell lung cancer, *LC* lung cancer, *NPC* nasopharyngeal carcinoma, *UC* urologic cancer, *HPBC* hepato-pancreato-biliary cancer, *GC* gastric cancer, *CRC* colorectal cancer, *RCC* renal cell carcinoma, *HNC* head and neck cancer, *N.A* not available

### Pooled HBVr rate among cancer patients receiving ICIs

**Fig. 2:**
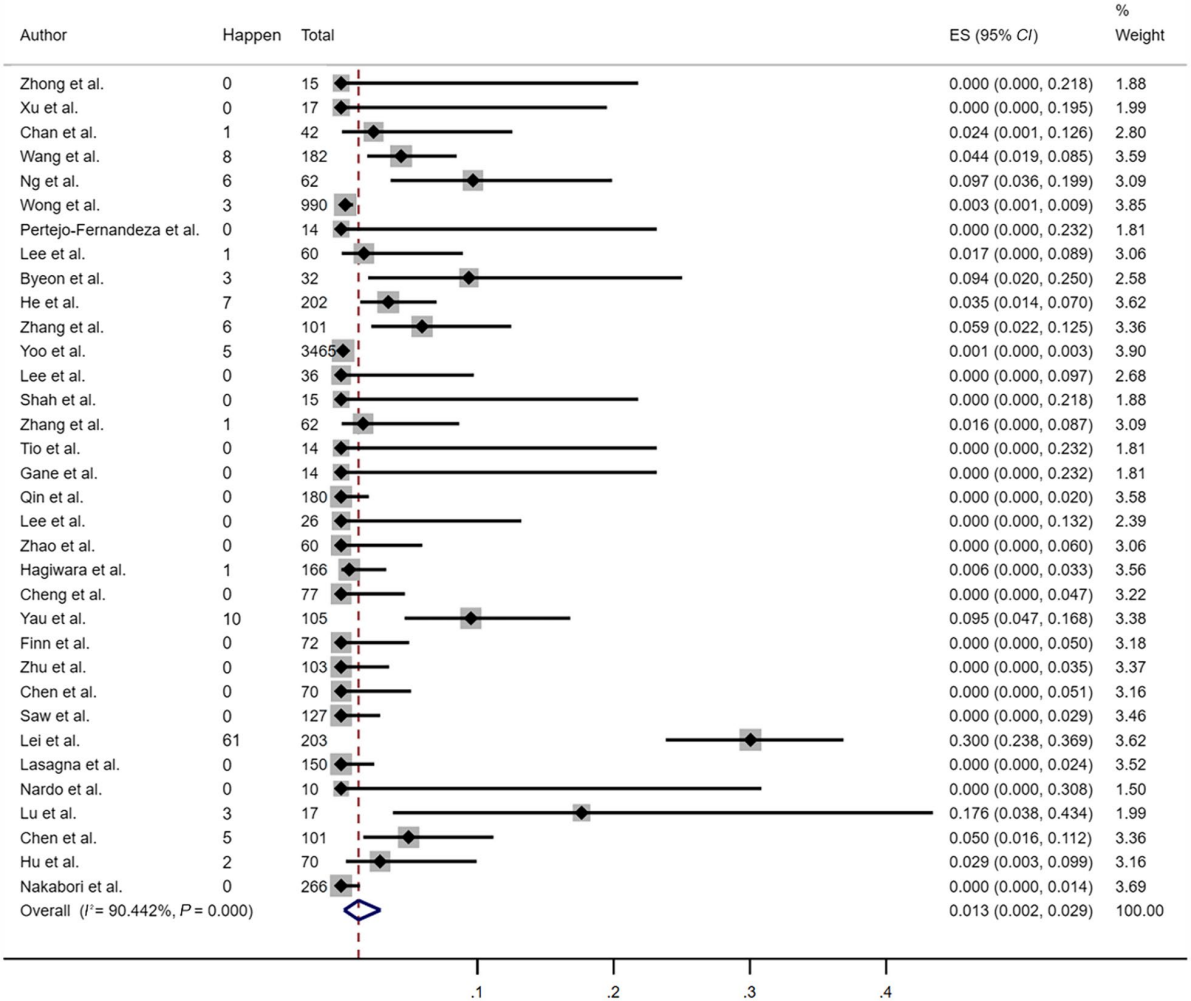
Pooled risk of HBVr among cancer patients with ICIs treatment. *HBVr* hepatitis B virus reactivation, *ICIs* immune checkpoint inhibitors, *ES* effect size, *CI* confidence interval

A total of 34 studies with a combined cohort of 7126 patients were analyzed to assess the incidence of HBVr in patients receiving ICI-based therapy for advanced cancer. As shown in Fig. 2, the pooled HBVr rate was 1.3% (123 reactivation cases out of 7126 patients). The risk estimates for HBVr varied from 0 to 30.0%, indicating considerable heterogeneity among the included studies (95% *CI*: 0.2–2.9%; *I*^2^ = 90.44%, *P* < 0.001).

### Subgroup analysis

In addition to the primary meta-analysis, various subgroup analyses were performed to investigate the sources of heterogeneity and the impact of multiple factors on the risk of HBVr in cancer patients receiving ICIs.

**Fig. 3:**
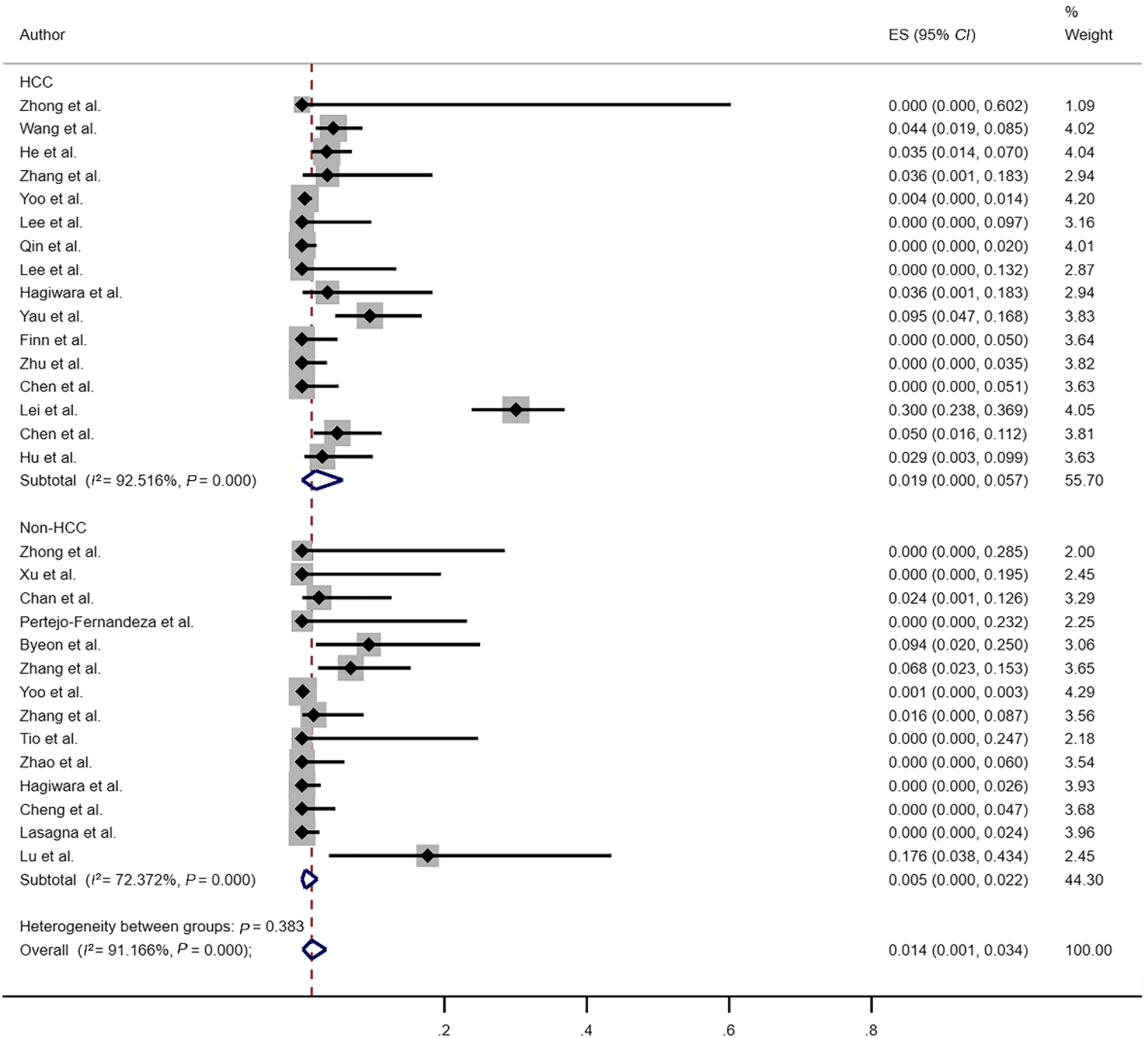
Risk of HBVr between HCC patients and non-HCC patients. *HBVr* hepatitis B virus reactivation, *HCC* hepatocellular carcinoma, *ES* effect size, *CI* confidence interval

In the subgroup analysis comparing HBVr rates between HCC and non-HCC patients (Fig. 3), the reactivation rates in HCC and non-HCC patients were 1.9% (95% *CI*: 0–5.7%; *I*^2^ = 92.52%, *P* < 0.001) and 0.5% (95% *CI*: 0–2.2%; *I*^2^ = 72.37%, *P* < 0.001), respectively. There was a difference in the reported reactivation rate between HCC and non-HCC patients with significant heterogeneity.

**Fig. 4:**
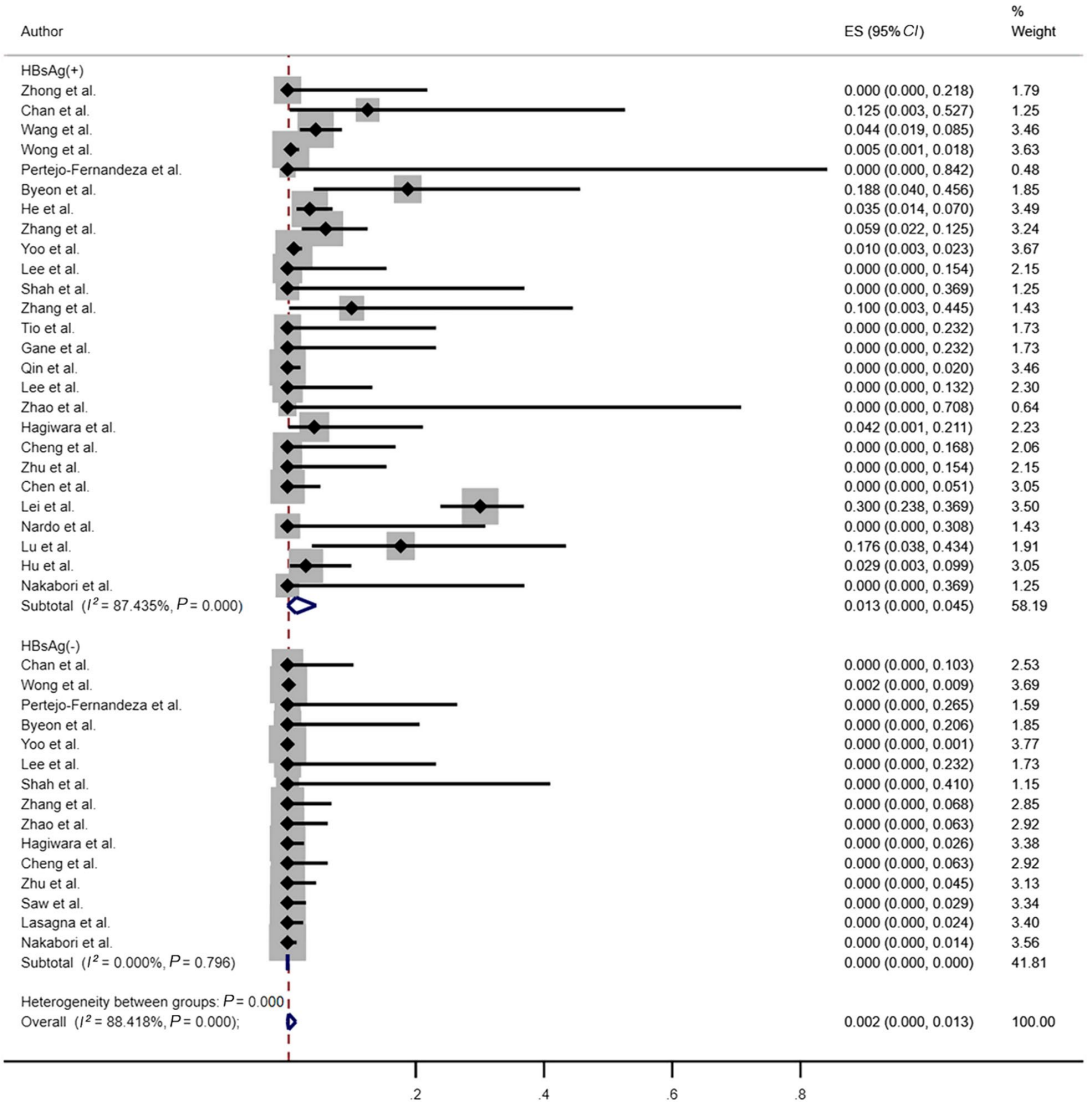
Risk of HBVr between HBsAg positive patients and HBsAg negative patients. *HBVr* hepatitis B virus reactivation, *HBsAg* hepatitis B surface antigen, *ES* effect size, *CI*: confidence interval

In the subgroup analysis comparing HBVr rates between HBsAg-positive and HBsAg-negative patients (Fig. 4), the reactivation rates in HBsAg-positive and HBsAg-negative patients were 1.3% (95% *CI*: 0–4.5%; *I*^2^ = 87.44%, *P* < 0.001) and 0 (95% *CI*: 0–0; *I*^2^ = 0, *P* = 0.796), respectively. Patients with positive HBsAg status had a higher risk of HBVr than those with negative HBsAg status.

**Fig. 5:**
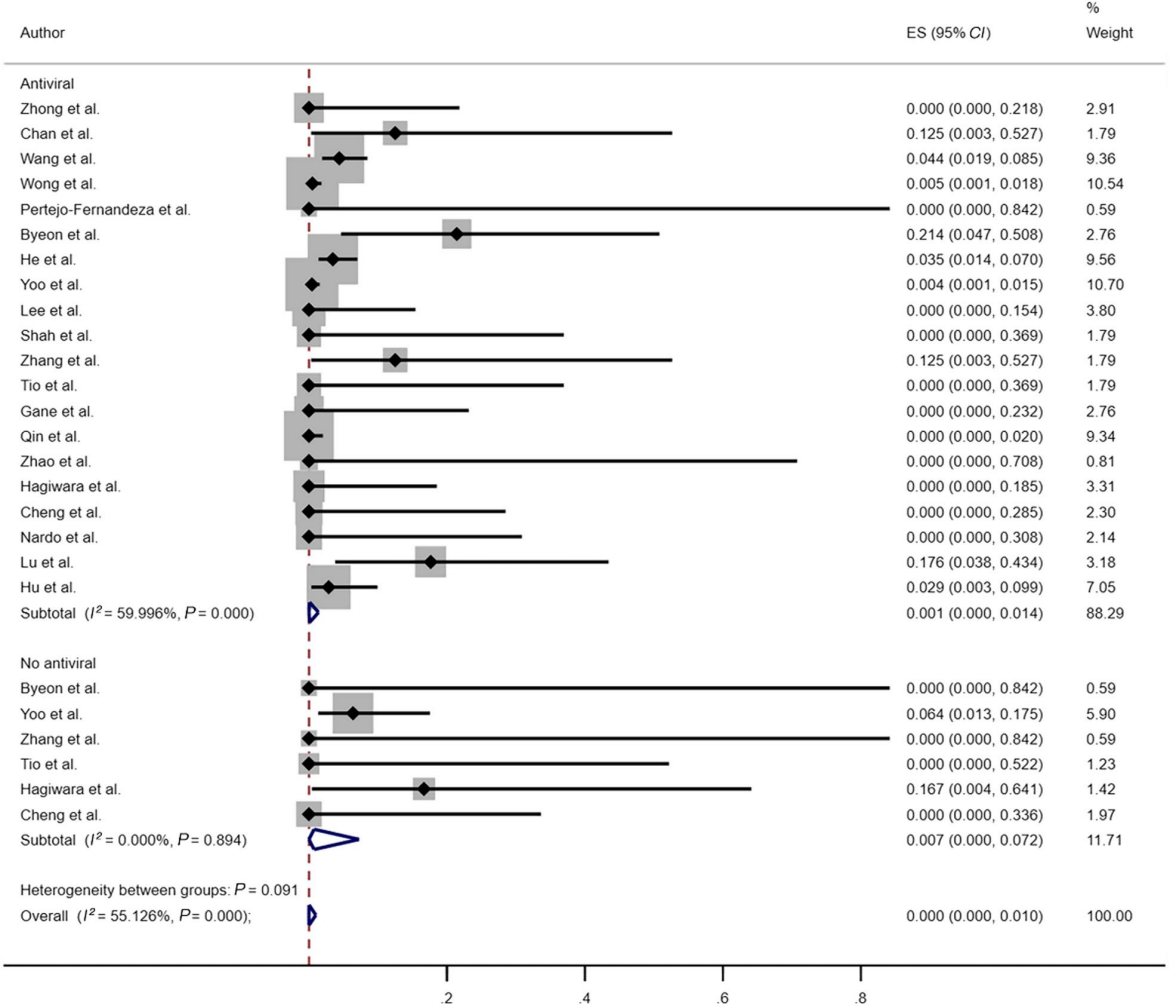
Risk of HBVr among HBsAg positive patients with or without antiviral prophylaxis. *HBVr* hepatitis B virus reactivation, *HBsAg* hepatitis B surface antigen, *ES* effect size, *CI* confidence interval

Our analysis included 34 studies, of which 21 studies reported cases of HBVr in HBsAg-positive cancer patients, as presented in Additional file 2: Table S2. Considering the potential risk of HBVr in HBsAg-positive individuals, we performed a subgroup analysis of antiviral therapy in this population. As shown in Fig. 5, in the comparison of HBVr rates between antiviral and no antiviral patients in HBsAg-positive patients, the reactivation rates in HBsAg-positive patients with or without antiviral prophylaxis were 0.1% (95%* CI*: 0–1.4%; *I*^2^ = 60.00%, *P* < 0.001) and 0.7% (95% *CI*: 0–7.2%; *I*^2^ = 0, *P* = 0.894), respectively. Patients on antiviral prophylaxis were found to have a lower risk of HBVr than those without antiviral prophylaxis.

**Fig. 6:**
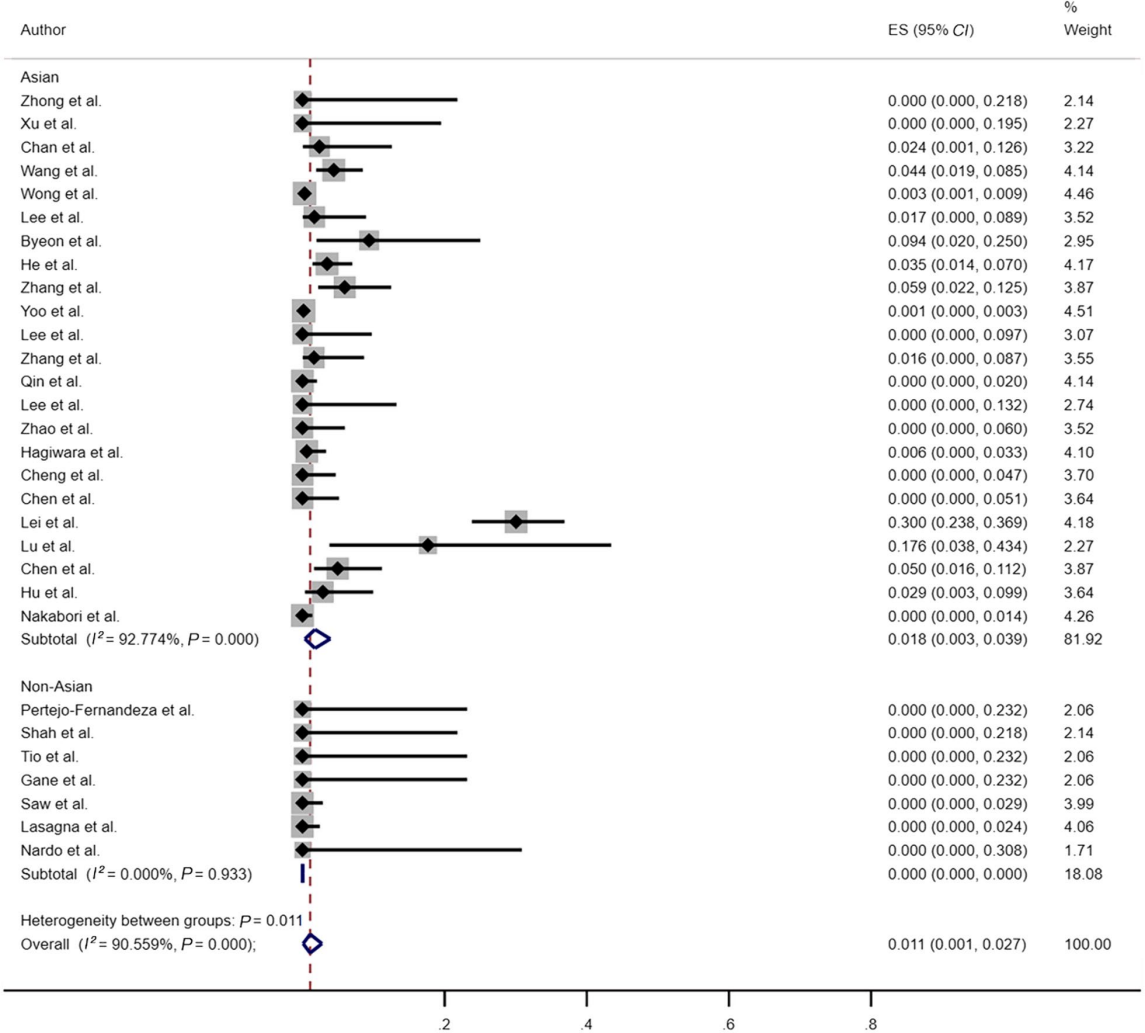
Risk of HBVr between Asian patients and non-Asian patients. *HBVr* hepatitis B virus reactivation, *ES* effect size, *CI* confidence interval

Given the regional variation in HBV patients distribution, we performed an analysis to determine whether the geographic origin of the included studies affected the reported rate of HBVr. (Fig. 6). Meta-analysis of the 23 Asian studies showed a pooled HBVr rate of 1.8% (95% *CI*: 0.3–3.9%; *I*^2^ = 92.77%, *P* < 0.001), whereas the reactivation rate in the 7 non-Asian studies was 0 (95%* CI*: 0–0; *I*^2^ = 0, *P* = 0.933). Our findings comparing HBVr rates between Asian and non-Asian patients indicate that the reported reactivation rate varied significantly between regions, with differences noted between Asian and non-Asian regions.

**Fig. 7:**
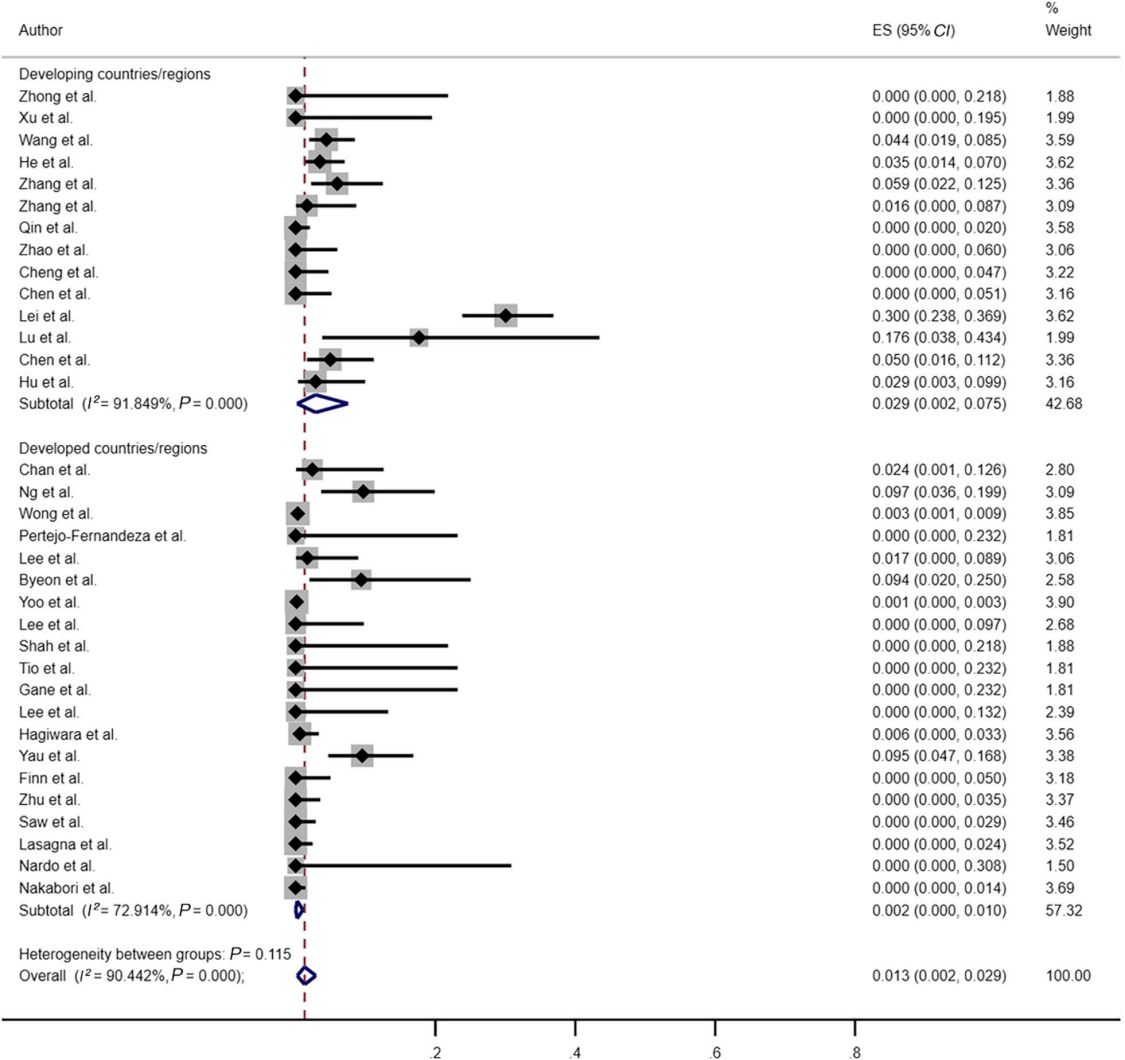
Risk of HBVr between developing vs developed countries. *HBVr* hepatitis B virus reactivation, *ES* effect size, *CI* confidence interval

To investigate the influence of socioeconomic factors on the variable HBVr rates reported in different studies, we further analyzed the included studies based on their level of regional economic development as classified by the International Monetary Fund (IMF) (https://data.imf.org/documents/WEOGroups.pdf, accessed on 10 May 2023) (Fig. 7) comparison of HBVr rates between developing and developed countries/regions. The meta-analysis of the 14 studies in developing countries/regions showed a pooled HBVr rate of 2.9% (95% *CI*: 0.2–7.5%; *I*^2^ = 91.85%, *P* < 0.001), whereas the reactivation rate in the 20 studies in developed countries/regions was 0.2% (95% *CI*: 0–1.0%; *I*^2^ = 72.91%, *P* < 0.001).

## Discussion

Our findings indicate that cancer patients exposed to ICIs have a measurable risk of HBV reactivation, which was estimated at 1.3%. ICI therapy can be considered close to a low risk factor, according to the threshold recommended by the AGA guideline for the prevention and management of HBVr, which defines an expected incidence of < 1% of cases as low risk. It is also lower than the reported spontaneous reactivation rate of HBV [21–25].

HBV can evade attack by HBV-specific immune cells and persist in the host through the presence of latent covalently closed circular DNA (cccDNA) or low-level replicating HBV following infection. This immune balance disruption can lead to HBVr. As ICIs target the immune evasion mechanisms of cancer cells, there is concern about the potential for ICIs to induce HBVr [26]. However, some reports indicate that ICIs may reduce the HBV viral load and maintain undetectable serum levels of HBV-DNA [26, 27]. Basic research in HBV carriers has shown that PD-1 is highly expressed on HBV-specific T cells and that PD-1 inhibitors may restore T-cell function. Additionally, studies have suggested that CTLA-4 monoclonal antibodies can block regulatory T-cell activity and restore the ability of follicular helper T cells to clear HBV. In a phase I clinical study, PD-1 blockade was found to restore HBV-specific immune responses in patients with chronic HBV infection [28]. However, the underlying mechanisms of the effect of ICIs on HBV require further exploration.

It is worth noting that among all HBsAg-positive patients, the pooled reactivation rate was 0.7% in the subgroup without antiviral drug intervention, while it was 0.1% in the subgroup with concomitant antiviral prophylaxis. These findings suggest that HBsAg-positive patients should not be excluded from eligibility to receive ICIs, as long as standardized antiviral prophylaxis is ensured throughout the entire course of therapy. Further research is necessary to determine the optimal antiviral prophylactic strategies for different patient populations.

Subgroup analysis revealed that patients from Asian regions or developing countries/regions had a higher rate of HBVr, which may be due to a higher prevalence of HBV carriers and lower socioeconomic status [29]. These findings are consistent with the results from the subgroup analysis, which confirmed that patients who were HBV carriers had a higher rate of HBVr than those who were HBsAg-negative. Due to the prolonged and resource-intensive nature of cancer and HBV treatment, particularly when expensive ICIs are involved in the antitumor regimen, patients in these countries may not be able to afford long-term monitoring and antiviral prophylaxis for HBV, even if recommended by health care professionals. However, studies are warranted with confounding factors controlled.

Subgroup analysis confirmed that patients with HCC have a higher risk of HBVr than those without HCC. However, due to insufficient data, subgroup analysis for solid tumors other than HCC was not performed. Further studies are needed to investigate whether patients with other types of solid tumors have different rates of HBVr when treated with ICIs.

This meta-analysis has several limitations that should be considered when interpreting the results. First, the study did not cover all types of ICIs and cancer types. Therefore, the findings may not be generalizable to all populations. Second, the majority of the included studies were retrospective, which may have led to high levels of selection bias. Third, significant heterogeneity among the existing studies made it difficult to accurately estimate the risk of HBVr in HBV carriers or patients with resolved hepatitis B who received ICI-based therapy for advanced cancer. Additionally, it is crucial to carefully consider individual patient characteristics and treatment regimens when evaluating the risk of HBVr in this population. Despite these limitations, given the severe situation surrounding hepatitis B prevention and control and the urgent need for evidence-based information, meta-analyses such as this are necessary. Further research is needed to expand upon these findings and better understand the risks associated with ICI-based therapy for advanced cancer in patients with hepatitis B.

## Conclusions

This study shows a measurable and potentially low risk of HBVr in patients with ICI treatment for advanced cancer. For those who are HBsAg-positive, prophylactic use of anti-HBV agents should be seriously considered before immunotherapy starts. Further large-scale prospective studies are warranted to confirm the findings.

### Supplementary Information

**Additional file 1: Table S1.** Full search strategies.

**Additional file 2: Table S2.** Subgroup data of HBsAg positive patients.

## Data Availability

Datasets are available through the corresponding author upon reasonable request.

## Abbreviations

ICIs: Immune checkpoint inhibitors
HBV: Hepatitis B virus
HBVr: Hepatitis B Virus reactivation
*CI*: Confidence intervals
ES: Effect size
HCC: Hepatocellular carcinoma
HBsAg: Hepatitis B surface antigen
PD-1: Programmed cell death protein 1
PD-L1: Programmed cell death ligand 1
CTLA-4: Cytotoxic T lymphocyte antigen 4
PRISMA: Preferred Reporting Items for Systematic Reviews and Meta-Analyses
HCV: Hepatitis C virus
HAV: Hepatitis A virus
HDV: Hepatitis D virus
HEV: Hepatitis E virus
NOS: Newcastle-Ottawa Scale
AASLD: American Association for the Study of Liver Diseases
ASCO: American Society of Clinical Oncology
IMF: International Monetary Fund
AGA: American Gastroenterological Association
cccDNA: Covalently closed circular DNA
TKIs: Tyrosine kinase inhibitors
MM: Malignant melanoma
GBM: Glioblastoma multiforme
UTUC: Upper tract urothelial carcinoma
NSCLC: Non-small cell lung cancer
LC: Lung cancer
NPC: Nasopharyngeal Carcinoma
UC: Urologic cancer
HPBC: Hepato-pancreato-biliary cancer
GC: Gastric cancer
CRC: Colorectal cancer
RCC: Renal cell carcinoma
HNC: Head and neck cancer
N.A.: Not available
