# Supplementary material for: Hepatitis B reactivation in cancer patients receiving immune checkpoint inhibitors: a systematic review and meta-analysis
Source: Infect Dis Poverty. 2023 Sep 22;12:87. doi: 10.1186/s40249-023-01128-6 (PMC10515058; doi:10.1186/s40249-023-01128-6)
Supplement: Supplementary file 1 — Additional file 1: Table S1. Full search strategies. [file 40249_2023_1128_MOESM1_ESM.docx]

**Table S1. Full search strategies**

| Database | Search Strategies | Number of Studies Identified |
| --- | --- | --- |
| PubMed | ((Immune Checkpoint Inhibitors[MeSH Terms] OR Immune Checkpoint Inhibitor* OR Immune Check point Inhibitor OR immune therapy OR immunotherapy OR cytotoxic T-lymphocyte associated antigen-4 OR CTLA-4 OR programmed cell death protein-1 OR programmed cell death protein OR programmed cell death-Ligand 1 OR PD-1 OR PD-L1 OR Ipilimumab OR tremelimumab OR nivolumab OR Opdivo OR pembrolizumab OR keytruda OR atezolizumab OR Tecentriq OR durvalumab OR Imfinzi OR avelumab OR Bavencio OR cemiplimab OR toripalimab OR Tuoyi OR sintilimab OR Camrelizumab OR SHR-1210 OR erica OR Tislelizumab OR MEDI4736 OR dostarlimab OR Penpulimab OR envafolimab OR KN035 OR Sugemalimab) AND (flare OR reactivation OR safety OR efficacy OR phase)) AND (Hepatitis B Virus OR HBV OR Chronic Hepatitis B Virus Infection OR Chronic Hepatitis B OR Hepatitis B Virus Inf ection, Chronic OR resolved hepatitis B OR hepatitis B surface antigen OR hepatitis B core antigen OR hepatitis B antibody OR HBsAg OR HBcAb OR HBV DNA* OR Hepatitis B Virus DNA*) AND (2000:2022[pdat]) | 2667 |
| Embase | ('immune checkpoint inhibitor'/exp OR 'immune checkpoint inhibitor*' OR 'immune check point inhibitor' OR 'immune therapy' OR 'immunotherapy' OR 'cytotoxic t-lymphocyte associated antigen-4' OR 'ctla-4' OR 'programmed cell death protein-1' OR 'programmed cell death protein' OR 'programmed cell death-ligand 1' OR 'pd-1' OR 'pd-l1' OR 'ipilimumab' OR 'tremelimumab' OR 'nivolumab' OR 'opdivo' OR 'pembrolizumab' OR 'keytruda' OR 'atezolizumab' OR 'tecentriq' OR 'durvalumab' OR 'imfinzi' OR 'avelumab' OR 'bavencio' OR 'cemiplimab' OR 'toripalimab' OR 'tuoyi' OR 'sintilimab' OR 'camrelizumab' OR 'shr-1210' OR 'erica' OR 'tislelizumab' OR 'medi4736' OR 'dostarlimab' OR 'penpulimab' OR 'envafolimab' OR 'kn035' OR 'sugemalimab') AND ('flare' OR 'reactivation' OR 'safety' OR 'efficacy' OR 'phase') AND ('hepatitis b virus'/exp OR 'hbv' OR 'chronic hepatitis b virus infection' OR 'chronic hepatitis b' OR 'hepatitis b virus inf ection, chronic' OR 'resolved hepatitis b' OR 'hepatitis b surface antigen' OR 'hepatitis b core antigen' OR 'hepatitis b antibody' OR 'hbsag' OR 'hbcab' OR 'hbv dna*' OR 'hepatitis b virus dna*') AND [2000-2022]/py | 1403 |
| Web of Science | ((TS=(Immune Checkpoint Inhibitor* OR Immune Check point Inhibitor OR immune therapy OR immunotherapy OR cytotoxic T-lymphocyte associated antigen-4 OR CTLA-4 OR programmed cell death protein-1 OR programmed cell death protein OR programmed cell death-Ligand 1 OR PD-1 OR PD-L1 OR Ipilimumab OR tremelimumab OR nivolumab OR Opdivo OR pembrolizumab OR keytruda OR atezolizumab OR Tecentriq OR durvalumab OR Imfinzi OR avelumab OR Bavencio OR cemiplimab OR toripalimab OR Tuoyi OR sintilimab OR Camrelizumab OR SHR-1210 OR Tislelizumab OR MEDI4736 OR dostarlimab OR Penpulimab OR Cyperizumab OR envafolimab OR KN035 OR Sugemalimab)) AND TS=(flare OR reactivation OR safety OR efficacy OR phase)) AND TS=(Hepatitis B Virus OR HBV OR Chronic Hepatitis B Virus Infection OR Chronic Hepatitis B OR Hepatitis B Virus Inf ection, Chronic OR resolved hepatitis B OR hepatitis B surface antigen OR hepatitis B core antigen OR hepatitis B antibody OR HBsAg OR HBcAb OR HBV DNA* OR Hepatitis B Virus DNA*) | 5838 |
| Cochrane Library | Immune Checkpoint Inhibitor* OR Immune Check point Inhibitor OR immune therapy OR immunotherapy OR cytotoxic T-lymphocyte associated antigen-4 OR CTLA-4 OR programmed cell death protein-1 OR programmed cell death protein OR programmed cell death-Ligand 1 OR PD-1 OR PD-L1 OR Ipilimumab OR tremelimumab OR nivolumab OR Opdivo OR pembrolizumab OR keytruda OR atezolizumab OR Tecentriq OR durvalumab OR Imfinzi OR avelumab OR Bavencio OR cemiplimab OR toripalimab OR Tuoyi OR sintilimab OR Camrelizumab OR SHR-1210 OR Tislelizumab OR MEDI4736 OR dostarlimab OR Penpulimab OR Cyperizumab OR envafolimab OR KN035 OR Sugemalimab in All Text AND flare OR reactivation OR safety OR efficacy OR phase in All Text AND Hepatitis B Virus OR HBV OR Chronic Hepatitis B Virus Infection OR Chronic Hepatitis B OR Hepatitis B Virus Inf ection, Chronic OR resolved hepatitis B OR hepatitis B surface antigen OR hepatitis B core antigen OR hepatitis B antibody OR HBsAg OR HBcAb OR HBV DNA* OR Hepatitis B Virus DNA* in All Text - with Cochrane Library publication date Between Jan 2000 and Jan 2022 (Word variations have been searched) | 955 |
| CNKI | (Yervoy OR Opdivo OR Keytruda OR Tecentriq OR durvalumab OR Imfinzi OR Bavencio) OR (Tislelizumab OR dostarlimab OR Penpulimab OR envafolimab OR Sugemalimab OR Serplulimab) OR (immune checkpoint inhibitor OR cytotoxic T lymphocyte associated antigen OR CTLA-4 OR programmed cell death protein OR programmed cell death receptor OR Ipilimumab OR Tremelimumab OR Tecillimumab OR nivolumab OR pembrolizumab OR atezolizumab OR durvalumab OR sintilimab OR Camrelizumab) OR CTL-4 OR PD-1 PR PD-L1 AND (hepatitis B OR hepatitis B antibody OR hepatitis B antigen OR hepatitis B virus OR hepatitis B surface antigen) OR HBsAg OR HBV DNA and Date:2000-* | 366 |
| SinoMed | ((hepatitis B OR hepatitis B antibody OR hepatitis B antigen OR hepatitis B virus OR hepatitis B surface antigen OR HBsAg OR HBV DNA OR safety) and (immune checkpoint inhibitor OR cytotoxic T lymphocyte associated antigen OR CTLA-4 OR programmed cell death protein OR programmed cell death receptor OR PD-1 OR PD-L1 OR Ipilimumab OR Yervoy OR Tremelimumab OR Tecillimumab OR nivolumab OR Opdivo OR pembrolizumab OR keytruda OR atezolizumab OR Tecentriq OR durvalumab OR Imfinzi OR avelumab OR Bavencio OR cemiplimab OR toripalimab OR Tuoyi OR sintilimab OR Camrelizumab OR Tislelizumab OR dostarlimab OR Penpulimab OR envafolimab OR Sugemalimab OR Serplulimab)) and Date:2000-* | 245 |
| Wanfang | ((hepatitis B OR hepatitis B antibody OR hepatitis B antigen OR hepatitis B virus OR hepatitis B surface antigen OR HBsAg OR HBV DNA OR safety) and (immune checkpoint inhibitor OR cytotoxic T lymphocyte associated antigen OR CTLA-4 OR programmed cell death protein OR programmed cell death receptor OR PD-1 OR PD-L1 OR Ipilimumab OR Yervoy OR Tremelimumab OR Tecillimumab OR nivolumab OR Opdivo OR pembrolizumab OR keytruda OR atezolizumab OR Tecentriq OR durvalumab OR Imfinzi OR avelumab OR Bavencio OR cemiplimab OR toripalimab OR Tuoyi OR sintilimab OR Camrelizumab OR Tislelizumab OR dostarlimab OR Penpulimab OR envafolimab OR Sugemalimab OR Serplulimab)) and Date:2000-* | 910 |
