# Supplementary material for: Hepatitis B reactivation in cancer patients receiving immune checkpoint inhibitors: a systematic review and meta-analysis
Source: Infect Dis Poverty. 2023 Sep 22;12:87. doi: 10.1186/s40249-023-01128-6 (PMC10515058; doi:10.1186/s40249-023-01128-6)
Supplement: Supplementary file 2 — Additional file 2: Table S2. Subgroup data of HBsAg positive patients. [file 40249_2023_1128_MOESM2_ESM.docx]

**Table S2 Subgroup data of HBsAg positive patients**

| **Number** | **First author (Publication year)** | **Study design** | **Study country** | **No. of HBsAg(+) patients** | **Antiviral**  **(YES vs NO)** | **HBVr rate**  **(Antiviral vs no Antiviral)** |
| --- | --- | --- | --- | --- | --- | --- |
| 1 | Martin Tio  (2018) | Retrospective | Australia | 14 | 8/6 | 0 vs 0 |
| 2 | Neil J. Shah  (2019) | Retrospective | United States | 8 | 8/0 | 0 vs 0 |
| 3 | Edward Gane  (2019) | Prospective | New Zealand | 14 | 14/0 | 0 vs 0 |
| 4 | AnaPertejo  (2020) | Retrospective | United States | 2 | 2/0 | 0 vs 0 |
| 5 | Gloria  (2020) | Retrospective | Singapore | 8 | 8/0 | 12.5% vs 0 |
| 6 | Seonggyu  (2020) | Retrospective | The Republic of Korea | 16 | 14/2 | 21.42% vs 0 |
| 7 | Shukui Qin  (2020) | Clinical trial | China | 180 | 180/0 | 0 vs 0 |
| 8 | Liting Zhong (2021) | Retrospective | China | 15 | 15/0 | 0 vs 0 |
| 9 | Kunyuan Wang  (2021) | Retrospective | China | 182 | 182/0 | 4.40% vs 0 |
| 10 | Grace  (2021) | Retrospective | China | 397 | 397/0 | 0.50% vs 0 |
| 11 | Min‑Ke He  (2021) | Retrospective | China | 202 | 202/0 | 3.47% vs 0 |
| 12 | Sun Yoo  (2021) | Retrospective | The Republic of Korea | 511 | 464/47 | 0.43% vs 6.38% |
| 13 | J.J.X. Lee  (2021) | Prospective | Singapore | 22 | 22/0 | 0 vs 0 |
| 14 | Xuanye Zhang  (2021) | Retrospective | China | 10 | 8/2 | 12.5% vs 0 |
| 15 | Zhiting Zhao  (2022) | Retrospective | China | 3 | 3/0 | 0 vs 0 |
| 16 | Satoru Hagiwara  (2022) | Retrospective | Japan | 24 | 18/6 | 0 vs 16.67% |
| 17 | Yi-Kan Cheng (2022) | Retrospective | China | 20 | 11/9 | 0 vs 0 |
| 18 | Shun Lu  (2022) | Clinical trial | China | 17 | 17/0 | 17.65% vs 0 |
| 19 | Xiaoyun Hu  (2022) | Retrospective | China | 70 | 70/0 | 2.86% vs 0 |
| 20 | Jin Lei  (2023) | Retrospective | China | 203 | 203/0 | 30.05% vs 0 |
| 21 | Mirella Nardo  (2023) | Retrospective | United States | 10 | 10/0 | 0 vs 0 |
